# The long road to protect infants against severe RSV lower respiratory tract illness

**DOI:** 10.12688/f1000research.18749.1

**Published:** 2019-05-02

**Authors:** Sofia Jares Baglivo, Fernando P Polack

**Affiliations:** 1 Fundacion INFANT, Buenos Aires, Argentina

**Keywords:** respiratory syncytial virus, vaccines, monoclonal antibodies, infants, pneumonia, asthma

## Abstract

Severe respiratory syncytial virus (RSV) lower respiratory tract illness (LRTI) in infants has proven challenging to prevent. In the last 50 years, conceptually different approaches failed to evolve into viable preventive alternatives for routine use. Inactivated RSV vaccine (that is, formalin-inactivated RSV) elicited severe LRTI in RSV-infected toddlers pre-immunized as infants; early purified F protein approaches in pregnant women failed to elicit sufficient immunity more than a decade ago; a second-generation monoclonal antibody (mAb) of high potency against the virus (that is, motavizumab) caused severe adverse reactions in the skin, and owing to lack of efficacy against RSV subgroup B, an extended half-life mAb targeting site V in the RSV fusion protein (that is, REG2222) did not meet its primary endpoint. In the meantime, two protein F vaccines failed to prevent medically attended LRTI in the elderly. However, palivizumab and the recent results of the Novavax maternal immunization trial with ResVax demonstrate that severe RSV LRTI can be prevented by mAb and by maternal immunization (at least to a certain extent). In fact, disease prevention may also decrease the rates of recurrent wheezing and all-cause pneumonia for at least 180 days. In this review, we discuss the history of RSV vaccine development, previous and current vaccine strategies undergoing evaluation, and recent information about disease burden and its implications for the effects of successful preventive strategies.

In 1962, following the isolation of the chimpanzee coryza agent—later renamed respiratory syncytial virus (RSV)—by Robert Chanock, the first vaccine against RSV was evaluated in 54 children in the US. RSV was inactivated using formalin (FIRSV) and 10 out of 21 vaccine recipients subsequently infected with the virus experienced severe lung disease. No children received placebo, and researchers attributed the observed severity of illness to an unusually bad season ^1^. Four years later, FIRSV was tested in four clinical trials in young children. Immunized infants developed an enhanced form of disease with severe wheezing and bronchopneumonia when infected with RSV ^1– 4^. Hospitalizations were frequent: up to 80% of infected vaccinees in one study ^1^. Two vaccinated toddlers—14 and 16 months of age—died when contracting RSV ^1^.

The pathogenesis of enhanced RSV disease (ERD) has been one of the main subjects of interest in the field in the last 50 years. In structural virology and immunology, every significant scientific advancement associated with the virus has been used to uncover new angles of this complex problem. In essence, two immune correlates are accepted as the main determinants of enhancement: the presence of low-avidity, non-protective antibodies elicited by immunization ^5– 7^ and a polarization of the immune response toward T helper 2 (Th2) in the respiratory tract after RSV infection ^8, 9^. Non-protective antibodies form pathogenic immune complexes in the lung that lead to complement activation and simultaneously fail to inhibit RSV replication. T helper lymphocytes polarized to type 2 responses after vaccination trigger an exuberant pulmonary infiltrate characterized by an excess in eosinophils and neutrophils. Recent observations in the field postulate a potential relationship between RSV F conformations in the vaccine and ERD ^10, 11^; new findings question whether route of immunization may be important for RSV vaccine responses ^12^; and improvements in our understanding of IgG memory B cells that have not undergone affinity maturation may help identify primed B-cell memory populations associated with disease enhancement ^13– 15^. Moreover, subpopulations of individuals with genetic mutations in molecules essential to B-cell maturation interrogate whether ERD could ever be observed in seropositive subjects ^16– 18^.

Given that memory T helper cells play a critical role in the severity of ERD presentations, approaches to RSV prophylaxis that rely on passive acquisition of antibody are unlikely to trigger adverse responses of extreme severity. Immunization of pregnant women to protect infants through transplacental transfer of antibody and administration of virus-specific monoclonal antibodies (mAbs) of extended half-life ^19, 20^ have undergone numerous phase 1 and phase 2 evaluations without evidence of ERD ^21– 23^. In addition, infant intranasal immunization with live-attenuated RSV vaccines (LAVs) mimics natural infection and has not been associated with ERD in phase 1 trials in seronegative subjects ^24^.

## Circumventing enhanced respiratory syncytial virus disease

After the adverse outcomes resulting from FIRSV immunization in 1967 ^1^, the RSV field focused on preventive strategies that excluded immunization of seronegative infants and young children with non-replicating RSV vaccines. The first LAV was developed at the National Institute of Allergy and Infectious Diseases (NIAID) in 1969. Evaluation of purified F protein (that is, PFP-2) in pregnant women failed to elicit sufficient antibody to achieve high levels of IgG transplacental transfer in the past ^25^. Passive transfer of sera enriched for anti-RSV F antibodies (RespiGam ^®^) ^26^ was followed by licensing of a humanized neutralizing mAb targeting site II of RSV F (that is, palivizumab) to prevent illness in infants at high risk for severe disease with 55% efficacy (tested in infants younger than 33 weeks of gestational age at birth) ^27^. This widely accepted approach to RSV disease prevention has limitations: it is costly for developing countries, requires monthly administration in young infants (often compromising compliance), and demands a good characterization of the circulating RSV strains and a clear understanding of the RSV season in tropical climates. In addition, results from the initial phase 3 clinical trial of palivizumab suggest that certain specific populations may be more challenging to protect than others. For instance, about 90% of all palivizumab treatment failures were heterozygotes for a loss-of-function single-nucleotide polymorphism in Asp299Gly or Thr399Ile (or both) of the pattern recognition receptor Toll-like receptor 4 (TLR4 ^+/−^) ^28^. These TLR4 ^+/−^ infants represent only about 10% of the US population and experience exaggerated Th2 responses in the respiratory tract during RSV infection ^29^. Also, in children from Navajo and Apache reservations, who are particularly susceptible to RSV ^30^, a second-generation high-affinity mAb against the virus did not prevent long-term recurrent wheezing (see below) despite reducing the rate of severe acute RSV disease ^31^. Thus, palivizumab remains the only licensed approach to RSV prevention today.

## Mapping severe respiratory syncytial virus lower respiratory tract illness and its long-term consequences

In the last decade, the field of RSV vaccines has witnessed an exponential growth of novel strategies and improvements in pre-existing approaches to tame this elusive virus ( https://path.azureedge.net/media/documents/RSV_Snapshot_2019_03_29_High_Resolution.pdf). These developments were coupled with a better understanding of disease biology and burden of illness worldwide. Studies from groups in Spain and the US identified a pre-fusion conformation in RSV F that exposed several protective epitopes and fostered development of new vaccines and mAb ^32^. Meanwhile, large global collaborative studies reported more than three million hospitalizations every year directly attributed to RSV along with about 118,000 deaths at the hospitals and in the community ^33^. Deaths in industrialized countries were infrequent and associated with chronic illness, from chronic lung disease and cyanotic congenital heart disease to neuromuscular or genetic disorders or both ^34^. In the developing world, deaths at the hospital often followed mechanical problems during medical care—that is, clinically significant pneumothoraxes (air leaks compromising oxygenation)—and secondary bacterial infections with Gram-positive cocci ^35^. Deaths in the community affected families in vulnerable socioeconomic situations who often did not visit medical facilities ^36^. Recent observations during the phase 3 evaluation of a maternal F nanoparticle vaccine to prevent RSV lower respiratory tract illness (LRTI) in young infants that failed to meet its primary endpoint may lead to revision of the estimated burden of RSV in the developing world. Interestingly, passively immunized infants had an unexpected reduction in all-cause medically significant (MS) LRTI, suggesting that RSV may be at play in circumstances we do not anticipate ^37^.

In addition, through prevention of RSV LRTI using palivizumab, a landmark study in premature babies showed that severe RSV LRTI also contributes to the inception of recurrent wheezing and potentially asthma in children ^38^. Other studies also examined the role of RSV in long-term wheezing and asthma through trials using mAb ^31, 39– 41^. The majority of these studies described a protective role against recurrent wheezing for RSV prevention using palivizumab in the first year of life ^38– 41^. Interestingly, a similar study with a high-affinity mAb against RSV (motavizumab) in healthy Native Americans born at term in Arizona had no effect on rates of medically attended wheezing in 1- to 3-year-old children despite preventing severe acute RSV LRTI ^31^.

Most of these studies monitoring wheezing reported a follow-up at the age of 6 years. In the Netherlands, palivizumab significantly reduced parent-reported asthma on the basis of different rates in infrequent wheezing (one to three episodes per year) when compared with placebo recipients ^42^. Physician-diagnosed asthma and lung function did not differ between groups ^42^. In Japan, palivizumab prophylaxis did not suppress the onset of atopic asthma but did reduce recurrent wheezing ^41^. In recent years, a group of investigators proposed a set of clinical criteria and lung function evaluations (forced oscillation technique at the age of 3 years and spirometry at the age of 6 years) for the evaluation of recurrent wheezing and asthma in clinical trials ^43^. Evidently, ongoing vaccine and mAb phase 3 studies represent a unique opportunity to answer these questions and settle the precise role of RSV in asthma inception. Yet asthma has proven to be a set of heterogeneous conditions with different molecular mechanisms of illness but common symptoms. These overlapping clinical phenotypes with distinct mechanisms of illness are known as asthma “endotypes” ^44^. Hence, it is probable that preventing severe RSV disease will modulate one or a few of these asthma “endotypes” but not influence others. (In fact, the lack of association between RSV and atopy is fairly well established ^45^.) Today, there is enough evidence to warrant a detailed evaluation of a role for RSV vaccines and mAbs in decreasing the burden of all-cause severe LRTI and pneumonia after RSV-specific antibodies become undetectable in serum ^35– 37^, having a greater-than-expected impact on mortality at the hospital and in the community given the aforementioned effects ^35– 37^, and modulating the burden of recurrent wheezing with potential long-term consequences in lung function and perhaps certain endotypes of asthma in school-age children ^39– 41^.

## Surge of approaches to prevent respiratory syncytial virus lower respiratory tract illness

There are three target populations that concentrate most efforts in RSV vaccine and mAb development. First, infants under 6 months of age constitute the main at-risk group. For them, two approaches are under consideration: passive transfer of protective antibody following maternal immunization and early administration of mAb of extended half-life. Second, in older children, active immunization with LAV or vectored vaccines is expected to extend protection through the first year(s) of life. Finally, adults older than 65 years may experience disease rates comparable to those elicited by influenza virus and are targeted for protection using active immunization with different constructs. Preventive strategies for the latter group are beyond the scope of this review, but it is important to note that two consecutive trials by Novavax and MedImmune failed to confer protection against medically attended RSV LRTI in the elderly with primary endpoint efficacies of −7.9% (95% confidence interval [CI] −84 to 37%) and −7.1% (95% CI −106.9 to 44.3%), respectively ^46– 48^.

Maternal immunization, after the widespread incorporation of influenza and pertussis vaccines to calendars, has been increasingly accepted as a strategy to provide protection to infants during their most vulnerable period. Maternal IgG antibodies are transferred across the placenta to fetal circulation through an active Fc receptor–mediated process during the third trimester of pregnancy and are expected to prevent severe disease inception in young infants. Recently, Novavax reported preliminary results from their evaluation of a pre-fusogenic nanoparticle vaccine in a randomized controlled trial in about 4,600 pregnant women immunized at a ratio of 2:1 between vaccinees and placebo recipients. This first-ever phase 3 study failed to meet its primary endpoint but exhibited 39.4% (95% CI 5.3 to 61.2%) efficacy at 90 days in reducing MS RSV LRTI globally with 44.4% (95% CI 19.6 to 61.5%) reduction in hospitalizations. The vaccine was safe with no observed difference in adverse events between vaccine and placebo recipients. Interestingly, results exhibited considerably more efficacy in South Africa than in the US: a pre-specified exploratory MS-RSV LRTI endpoint including additional cases retrieved from hospital chart reviews ranged from 57% (95% CI 32.7 to 72.5%) to −32.7% (95% CI −238.9 to 48.1%). Unfortunately, the study was not sufficiently powered to confidently ascertain whether these differences simply represent variations within the CI or respond to other mechanisms. Immunization more than 30 days before delivery enhanced the efficacy of the vaccine ^37^. Importantly, the Novavax RSV pre-fusogenic vaccine conferred protection against two additional pre-specific endpoints: 25.3% against all-cause LRTI and 39.1% against all-cause LRTI with significant hypoxemia (<92% O _2_ saturation) globally ^37^. Other maternal immunogens include a GlaxoSmithKline recombinant RSV protein F vaccine, engineered to preferentially maintain pre-fusion conformation (NCT02753413) ^19, 49^; a Pfizer subunit vaccine encoding a stabilized pre-fusion molecule also intended for the elderly (NCT03529773) ^21^; and the RSV F DS-Cav1 (NCT03049488) candidate developed by the NIAID ^50^. This last formulation engineered soluble site Ø–stabilized RSV F trimers (site Ø is present on pre-fusion F only) adjuvanted with alum.

Development of mAb against RSV has also faced considerable challenges in recent years. Motavizumab, a second-generation mAb of high potency derived from palivizumab by MedImmune using affinity maturation techniques, reduced by 87% (95% CI 79 to 92%) the incidence of hospitalization due to RSV and outpatient visits by 71% (95% CI 58 to 80%) in term infants not older than 6 months of age in Navajo and Apache reservations ^31^. However, the drug exhibited serious adverse events—including hypersensitivity or allergic events, erythema multiforme, and a self-resolving transient skin erythema—probably associated with the study product ^31^. Evaluation of suptavumab (REGN2222), an antibody of extended half-life developed by Regeneron against site V in RSV F, failed to meet its primary efficacy endpoint in preterm infants (NCT02325791) ^51^. Despite presenting a protective trend against medically attended infections caused by RSV subgroup A, an unexpected mutation in site V of RSV F in circulating RSV B viruses affected its overall performance.

MedImmune, the manufacturer of palivizumab, is now evaluating MEDI8897, timed to enter phase 3 trials in the coming year. MEDI8897 presents amino acid substitutions in the Fc region—Met252Tyr/Ser254Thr/Thr256Glu (YTE technology)—that augment binding to the histocompatibility complex class I-neonatal Fc receptor and extend its half-life to 85 to 117 days ^52– 55^. This attractive candidate received breakthrough therapy designation from the US Food and Drug Administration and aims to protect infants against RSV with a single injection. The mAb targets antigenic site Φ in the pre-fusion conformation of RSV F. Another mAb of extended half-life undergoing evaluation is MK-1654 from Merck (NCT03524118) targeting RSV F site IV ^56^.

For older children, there are several live-attenuated vaccines in clinical trials, all of them in phase 1. A candidate from NIAID-Sanofi with an NS2 non-structural protein deletion that reduces viral suppression of the interferon response and increases the innate immune response has advanced in parallel with a second NIAID-Sanofi candidate with a deletion in the RSV M2-2 protein attenuating viral replication while upregulating gene transcription and antigen expression (NCT02237209 and NCT02040831) ^57– 62^. These candidates appear mildly over- and under-attenuated, respectively ^61^. Vectored approaches for children include Ad26.RSV.preF using a human adenovirus 26 now in phase 2 trials in adults and 12- to 24-month-old RSV seropositive toddlers (NCT03303625) ^63, 64^ and ChAd155-RSV (NCT02927873) ^65^, a replication-incompetent chimpanzee adenovirus 155 now in phase 2 in seropositive children. This vector encodes the F, N, and M2-1 RSV proteins ^65^. MVA-BN-RSV from Bavarian Nordic uses modified vaccinia Ankara virus encoding F, G, N, and M2-1 antigens and has completed phase 2 in adults. Finally, the rBCG-N-hRSV vaccine (NCT03213405) ^66^ is a chimeric candidate using recombinant BCG expressing RSV N protein and is targeted for use in newborns. This BCG construct would allow simultaneous early protection against two serious ailments while inducing Th1 immunity, skewing responses away from the undesirable Th2 priming typical of ERD ^66, 67^. In addition, rBCG-N-hRSV is expected to elicit cellular immunity against RSV ^68^.

## Final considerations

Prevention of severe RSV LRTI has proven challenging for various approaches, but recent results from maternal immunization using ResVax ^®^ and the effectiveness of palivizumab demonstrate that conferring some degree of protection is possible. Numerous strategies are advancing through human trials. Studies in the next few years should contribute to better contextualize the benefits of each successful existing and upcoming intervention.
